# Continuous representation‐based reconstruction for computed tomography

**DOI:** 10.1002/mp.17849

**Published:** 2025-05-02

**Authors:** Minwoo Yu, Junhyun Ahn, Jongduk Baek

**Affiliations:** ^1^ Department of Artificial Intelligence Yonsei University Seoul South Korea; ^2^ School of Integrated Technology Yonsei University Seoul South Korea; ^3^ BareuneX Imaging Inc. Seoul South Korea

**Keywords:** computed tomography (CT) image, continuous image representation, sinogram squeezing, sinusoidal basis decoder

## Abstract

**Background:**

Computed tomography (CT) imaging has been developed to acquire a higher resolution image for detecting early‐stage lesions. However, the lack of spatial resolution of CT images is still a limitation to fully utilize the capabilities of display devices for radiologists.

**Purpose:**

This limitation can be addressed by improving the quality of the reconstructed image using super‐resolution (SR) techniques without changing data acquisition protocols. In particular, local implicit representation‐based techniques proposed in the field of low‐level computer vision have shown promising performance, but their integration into CT image reconstruction is limited by considerable memory and runtime requirements due to excessive input data size.

**Methods:**

To address these limitations, we propose a continuous image representation‐based CT image reconstruction (CRET) structure. Our CRET ensures fast and memory‐efficient reconstruction for the specific region of interest (ROI) image by adapting our proposed sinogram squeezing and decoding via a set of sinusoidal basis functions. Furthermore, post‐restoration step can be employed to mitigate residual artifacts and blurring effects, leading to improve image quality.

**Results:**

Our proposed method shows superior image quality than other local implicit representation methods and can be further improved with additional post‐processing. In addition proposed structure achieves superior performance in terms of anthropomorphic observer model evaluation compared to conventional techniques. This results highlights that CRET can be used to improve diagnostic capabilities by setting the reconstruction resolution higher than the ground truth images in training.

**Conclusions:**

Our proposed CRET method offers a promising solution for improving CT image resolution while addressing excessive memory and runtime consumption. The source code of our proposed CRET is available at https://github.com/minwoo‐yu/CRET.

## 1 INTRODUCTION

Computed tomography (CT) imaging system is an imaging modality to reconstruct images using x‐ray projection data acquired from multiple angles.^1^ Since internal anatomical information can be obtained without applying an invasive injury, it is widely used in the field of medical imaging. Due to technological advances, such as the increased number of detector rows with a smaller detector pixel pitch, the spatial resolution of CT imaging systems has been significantly improved. As a result, the current clinical CT imaging systems are capable of producing reconstructed images with a submillimeter resolution, accompanying 512×512 image array size.^2^ Despite these advancements, the display devices used by radiologists are capable of visualizing higher resolution (e.g., up to 4200×2800 pixels on displays from Barco) than currently achievable with CT imaging. Consequently, radiologists cannot utilize the full potential of advanced high‐resolution display devices, making an accurate diagnosis. This limitation is particularly evident when zoomed region‐of‐interest (ROI) images are used for the diagnosis of detailed anatomical structures. The magnified array size of the ROI image exacerbates blocky artifacts, thereby hindering the detection of small lesions measuring only a few millimeters.^3^ In addition, although higher resolution image is achievable using a detector with submillimeter resolution, it requires a sufficiently high x‐ray dose. As a result, the difficulty in increasing the radiation dose per detector leads to increased noise levels, which are concentrated in the high‐frequency components and hinder the diagnosis of fine details.^4^, ^5^, ^6^ Detector binning can help mitigate this problem by combining signals from adjacent detectors. Also, it is utilized in multiple clinical applications, including reducing x‐ray scanning time in breast CT imaging^7^, ^8^ and mitigating the charge‐sharing effect in photon‐counting detectors (PCDs).^9^, ^10^ However, as a trade‐off, the detector binning leads to blurring in the reconstructed images, which results in decreased diagnostic performance for small lesions. Therefore, image processing techniques are required to overcome both the limitations in spatial resolution arising from the finite detector pixel pitch size and the degradation of image quality caused by noise and blurring effects.

To address the limited resolution performance and degradation by noise and blurring, various deep learning‐based super‐resolution (DL‐SR) techniques have been developed. These approaches primarily focus on modifying network architectures to improve resolution while simultaneously mitigating image quality degradation arising from noise and blurring effects. However, most conventional SR techniques focus on training models for a fixed upsampling scale, and thus a separate network should be trained for a different upsampling scale. It lacks flexibility, limits the ability to adjust the resolution of the output image and struggles to achieve resolutions exceeding the ground truth (GT) images used in training. These limitations make it difficult to optimize spatial resolution for various diagnostic scenarios and conditions (e.g., acquisition settings like detector binning scale, type of display device used for diagnostics). Thus, it is desirable to develop a novel upsampling structure capable of performing SR at arbitrary upsampling scales using a unified model. For instance, arbitrary scale super‐resolution (ArbSR) structures that have been proposed in the field of low‐level computer vision can be utilized.^11^, ^12^, ^13^, ^14^ Among ArbSR structures, the local implicit image function (LIIF)^11^ inspired from implicit neural representation (INR) has demonstrated promising performance. LIIF extracts the feature from the input image via the encoder and then performs image representation using the feature and relative coordinate as input to the multilayer perceptron (MLP) decoder.

By applying ArbSR techniques to CT imaging, the spatial resolution can be flexibly adjusted and the degradation due to noise and blurring can be mitigated. For implementation in detail, LIIF‐based architectures can be integrated into the filtered back‐projection (FBP) reconstruction algorithm. This integrated approach is analogous to the structure proposed by Würfl et al.,^15^ which involves processing in the sinogram domain using a neural network, followed by back‐projection. However, by integrating the decoding of LIIF into the back‐projection module, it demonstrates superior flexibility. This allows for effective adaptation to varying detector binning scales compared to using a simple back‐projection module. However, the required computations and memory increase exponentially compared to when the LIIF‐based architecture was used for photographic imaging, making its integration into FBP impractical. This inefficiency arises from two main factors: the computing resource consumption of the decoder increases linearly with the multiple of the number of projection views, and the utilization of the sinogram data is inefficient for the reconstruction of the target ROI image.

**FIGURE 1:**
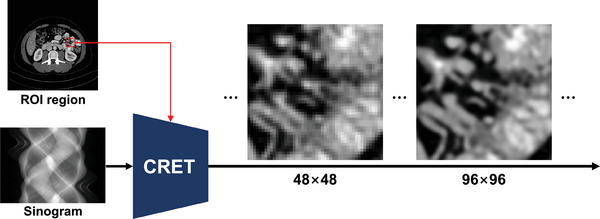
Continuous image representation‐based CT image reconstruction (CRET) is a technique that enables higher spatial resolution in CT imaging while mitigating the quality degradation caused by noise and blurring. CT, computed tomography.

To address this inefficiency, we propose a continuous representation‐based reconstruction technique for CT images (CRET) that improves the efficiency of computational resource over conventional local implicit representation‐based methods. CRET is optimized for reconstructing patch images of a specific ROI to compensate for the lack of spatial resolution when radiologists utilize them for diagnosis, as shown in Figure 1. We confirmed that CRET is more efficient in terms of time and memory consumption than conventional methods, which allow real‐time visualization of reconstructed images. Moreover, CRET shows superior image quality on multiple evaluation metrics, including a lesion detection task.

To summarize, our main contributions are as follows:

1. We propose a continuous representation‐based technique for reconstructing CT images. Our proposed structure is a unified model that allows flexible adjustment regardless of the binning scale and reconstruction resolution while mitigating blurring effects and noise issues.
2. Our proposed structure comprises two steps: a reconstruction step leveraging sinogram domain data to enable a continuous representation, and a restoration step using image domain data to further improve image quality. Notably, the restoration step is plug‐and‐playable, enabling radiologists to selectively apply it as needed.
3. Our structure achieves approximately over four‐fold reductions in both time and memory consumption compared to conventional local implicit representation‐based methods. In addition, we have confirmed that our structure shows superior image quality over conventional methods through a multifaceted evaluation.

## 2 RELATED WORKS

### 2.1 INR

INR is a technique that uses MLP with coordinate input to represent a specific signal and has recently achieved high performance using sine activation functions, positional encoding, and extracting Fourier features.^16^, ^17^, ^18^ This concept has been applied to achieve high performance in various areas such as 3D rendering,^17^, ^19^, ^20^ CT reconstruction under sparse‐view or limited‐angle conditions,^21^, ^22^, ^23^, ^24^, ^25^ and SR,^12^, ^13^, ^26^ and continuous representation of arbitrary discrete signal is achievable. In this study, we take advantage of the continuous representation capabilities of INR to reconstruct CT images.

### 2.2 ArbSR

Since the efficient sub‐pixel convolutional neural network (ESPCN)^27^ proposed an upsampling module that conducts upsampling through pixel shuffle layers, most SR architectures still conduct upsampling via pixel shuffle layers. However, this pixel shuffle upsampling module has a notable limitation: it only works for a fixed integer scale, and thus a separate model should be trained for a different upscaling factor. To resolve this issue, ArbSR methods have been proposed, which allow upsampling for arbitrary scales with a single model.^11^, ^12^, ^13^, ^14^, ^26^, ^28^, ^29^ In particular, LIIF^11^ performs ArbSR via a local INR technique, which enables the estimation of high‐quality images even when the upsampling scale exceeds the training scale range. The architecture of LIIF comprises two components: an encoder $E_{\phi }$, which extracts features from input image $I^{LR}$, and a decoder $f_{\theta }$, responsible for represent image pixel value $I(x_{q})$ corresponding to the coordinate $x_{q}$. Specifically, decoder performs a linear combination of the features $z_{t}^{*}$ corresponding to the four closest coordinates $x_{t}$ as follows:

(1) $$\begin{array}{c} \begin{array}{ccc} I(x_{q}) & = & \sum_{t\in \{00,01,10,11\}}\frac{S_{t}}{S}\cdot f_{\theta }\left(z_{t}^{*},x_{q}-x_{t}\right),\\ & & \text{where}\;z^{*}=E_{\phi }\left(I^{LR}\right) \end{array} \end{array}$$

where $S=\sum_{t\in \{00,01,10,11\}}S_{t}$ is the sum of rectangle areas between $x_{q}$ and $x_{t}$. Following the proposal of LIIF structure, multiple subsequent structures have been proposed for higher image quality than LIIF^12^, ^13^, ^26^ in the field of low‐level computer vision.

### 2.3 CT image reconstruction and applying SR techniques

Unlike photographic imaging, CT imaging involves a reconstruction process using a sinogram, and the commonly used FBP algorithm ensures a fast reconstruction time.^30^ However, the FBP algorithm is limited by its spatial resolution due to the finite detector pixel pitch size and it struggles with vulnerability to noise and blurring degradation factors during reconstruction.^7^ To overcome these limitations, applying DL‐SR techniques has emerged as promising solutions.^31^, ^32^, ^33^, ^34^ However, applying DL‐SR techniques directly to CT images improves visual quality as a post‐processing step without modifying the underlying reconstruction process. This results in sub‐optimal performance because there is no improvement to the fundamental reconstruction process of CT imaging. Therefore, methodologies leveraging sinogram data have been proposed^35^, ^36^ and demonstrated superior performance compared to approaches solely relying on image data.^37^ Building upon this foundation, our proposed CRET also utilizes sinogram domain data as input and integrates a decoder into the back‐projection.

## 3 METHODOLOGY

### 3.1 Incorporating local implicit representation in FBP

**FIGURE 2:**
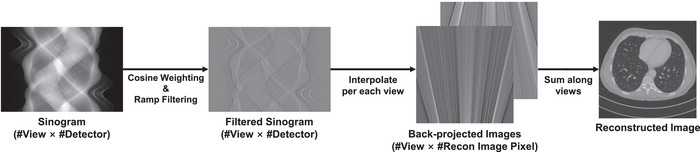
Procedure of FBP algorithm for FBCT imaging. FBP, filtered back‐projection; FBCT, fan‐beam CT.

**FIGURE 3:**
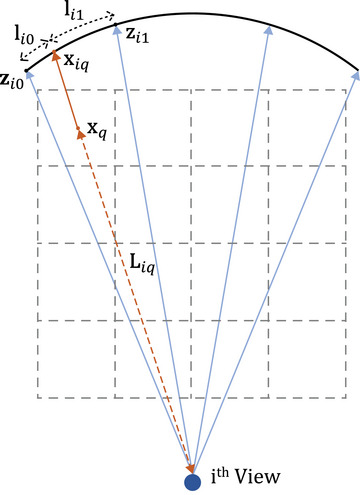
Illustration of back‐projection for FBCT geometry. 1D interpolation is performed on the back‐projection for the *i*th view. FBCT, fan‐beam CT.

In general, fan‐beam CT (FBCT) performs scanning by projecting x‐rays in a fan‐shaped pattern from a single x‐ray source.^1^ By using this scanned sinogram data p and its filtered sinogram z, image reconstruction is performed, and reconstructed image $\hat{y}$ can be mathematically expressed as follows:

(2) $$\hat{y}=A_{\mathrm{fb}}^{T}{\mathrm{CW}}_{\cos}p=A_{\mathrm{fb}}^{T}z$$

where $A_{\mathrm{fb}}^{T}$ denotes back‐projection for FBCT geometry, and C and where $W_{\cos}$ denotes filtering and cosine weighting, respectively. This overall image reconstruction pipeline can be illustrated as shown in Figure 2. In detail, the back‐projection operation $A_{\mathrm{fb}}^{T}$ can be expressed as performing an interpolation for each projection view. If linear interpolation is used for back‐projection, estimating the pixel value $\hat{y}\left(x_{q}\right)$ of the reconstructed image for an arbitrary coordinate $x_{q}$ can be illustrated as shown in Figure 3 and expressed as follows:

(3) $$\hat{y}\left(x_{q}\right)=\sum_{i=1}^{N}\frac{1}{L_{iq}^{2}}\cdot \frac{1}{l_{i}}\left(l_{i0}\cdot z_{i1}+l_{i1}\cdot z_{i0}\right),\;\text{where}\;l_{i}=l_{i0}+l_{i1}$$

where $z_{i0}$ and $z_{i1}$ are the values of the filtered sinogram nearest to the coordinate $x_{q}$ in the *i*th view projection. Also, $x_{iq}$ is the coordinate value corresponding to $x_{q}$ on the projected data, while $l_{i0}$, $l_{i1}$, and $L_{iq}$ are the distances between $x_{iq}$ to $z_{i0}$, $x_{iq}$ to $z_{i1}$, and $x_{q}$ to x‐ray source, respectively.

Similarly, local implicit representation can be adapted into FBP for CT image reconstruction, and Equation (3) can be reformulated as follows:

(4) $$\begin{array}{c} \begin{array}{cc} \hat{y}\left(x_{q}\right)= & \sum_{i=1}^{N}\frac{1}{L_{iq}^{2}}\cdot \frac{1}{l_{i}}\left(l_{i0}\cdot f_{\theta }\left(z_{i1}^{*},x_{iq}-x_{i1}\right)\right.\\ & +\left.l_{i1}\cdot f_{\theta }\left(z_{i0}^{*},x_{iq}-x_{i0}\right)\right),\text{where}\;z^{*}=E_{\phi }\left(z\right) \end{array} \end{array}$$

where z denotes the filtered sinogram. By integrating the decoder into the part of the back‐projection that performs the interpolation, high image quality can be expected even if the reconstruction resolution differs from the GT image. However, this direct application of the LIIF‐based structure on the filtered sinogram requires a huge amount of time and memory consumption in both the encoder and decoder. To reduce computational resource requirements, photographic imaging utilizes cropped low‐resolution (LR) images as input data corresponding to the specific ROI to be targeted. On the contrary, in the case of CT imaging, simply cropping the sinogram into a rectangular patch cannot be used for ROI image reconstruction because the area of projection data that contributes to the ROI reconstruction is not confined to a rectangular region, but has an irregular shape.

**FIGURE 4:**
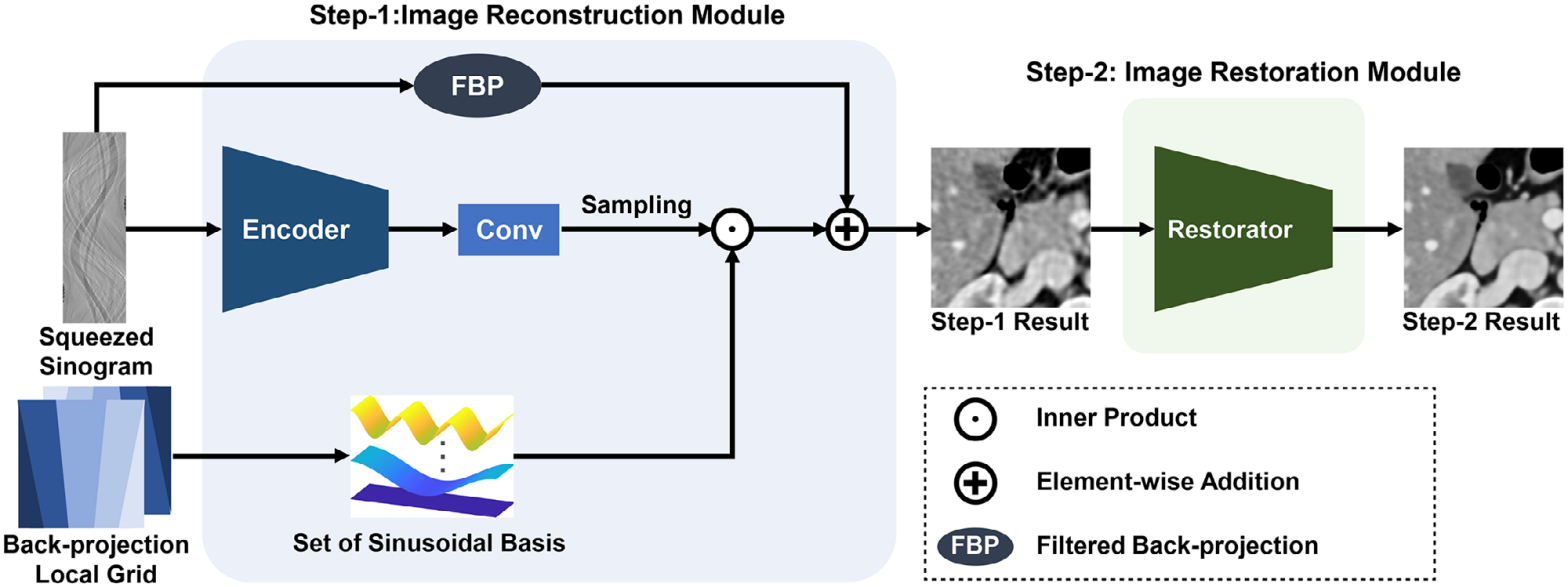
Illustration of the proposed CRET framework. CRET consists of two modules: an image reconstruction module that performs image reconstruction using the continuous representation technique, and an image restoration module that corrects residual artifacts and improves sharpness.

Next, there are also inefficiencies in the decoding process. For instance, when performing upsampling on a photographic image using Equation (1) and targeting an output image resolution as H×W with a number of feature maps as F, the decoder input data size is F×H×W. On the other hand, in CT image reconstruction, features sampled from the sinogram via back‐projection grids with dimensions N×F×H×W are employed as a decoder input, where N represents the number of projection views. Despite reconstructing a target image with a resolution comparable to photographic images, the significantly increased input data size renders decoding impractical due to excessive computational resource consumption. Therefore, we propose CRET, a structure optimized for CT image reconstruction that addresses inefficiencies in both the encoder and decoder. The overall pipeline of the proposed CRET is illustrated in Figure 4.

### 3.2 Step‐1: Reconstruction module

The first module of our proposed CRET integrates FBP with a continuous representation technique. This module corresponds to the blue shaded part in Figure 4, and it can be expressed mathematically by reformulating Equation (3) as follows:

(5) $$\begin{array}{c} \begin{array}{cc} \hat{y}\left(x_{q}\right)= & \sum_{i=1}^{N}\frac{1}{L_{iq}^{2}}\cdot \frac{1}{l_{i}}\left(l_{i0}\cdot f\left(z_{i1}^{*},x_{iq}-x_{i1}\right)\right.\\ & +\left.l_{i1}\cdot f\left(z_{i0}^{*},x_{iq}-x_{i0}\right)+l_{i0}\cdot z_{i1}+l_{i1}\cdot z_{i0}\right),\\ & \;\text{where}\;z^{*}=A_{\gamma }(E_{\phi }\left(S\left(z\right)\right) \end{array} \end{array}$$

where $A_{\gamma }$ denotes a convolution layer, which is added to equalize the number of channels in the feature and the number of decoder bases. S is the sinogram squeezing and unfolding proposed in this work, which reduces the input data size of the encoder. Here, f denotes a decoder similar to $f_{\theta }$, but instead of an MLP‐based decoder, it uses a trainable parameter‐free sinusoidal decoder inspired by orthogonal positional encoding super‐resolution (OPE‐SR).^14^

#### 3.2.1 Sinogram squeezing

**FIGURE 5:**
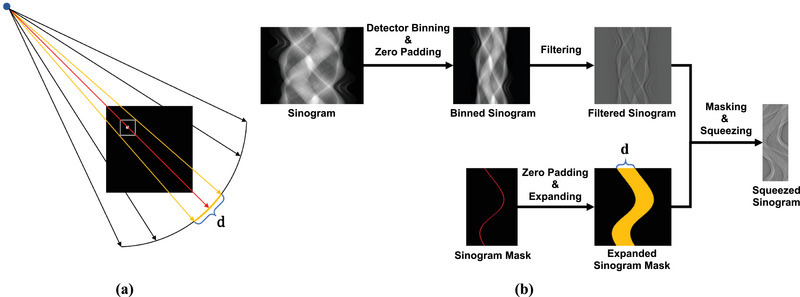
Illustration of the sinogram squeezing to reduce encoding time and memory consumption. (a): Acquisition of a sinogram mask corresponding to the ROI image reconstruction at a specific view angle. (b): The overall pipeline of sinogram squeezing. ROI, region of interest.

Sinogram squeezing technique removes the unnecessary sinogram components for the ROI image reconstruction and converts the remaining parts into a rectangular shape. First, locate the index in the projection data that corresponds to the center point of the ROI image to be reconstructed via forward projection. This corresponding index is shown as a red line on the sinogram mask in Figure 5. This red line represents the sinogram component required for the reconstruction of a specific point. Next, to extend reconstruction to an arbitrary ROI encompassing a specific point, additional sinogram components within the red line boundary are required. Even if the desired range surrounding the red line depends on the shape and position of the ROI, this complexity can be simplified by fixing it as a maximum range d. Accordingly, the sinogram mask is expanded by d pixels centered on the index, resulting expanded sinogram mask as illustrated in the orange region of Figure 5. After that, the filtered sinogram component corresponding to this expanded sinogram mask is recombined into a squeezed sinogram with a resolution of N×d. Note that zero padding is performed on the sinogram and sinogram mask prior to the expanding index, as the expanded index value may be out of the range of the sinogram index. By removing unnecessary data components for ROI image reconstruction using the squeezed sinogram as the input for the encoder, time and memory consumption can be reduced for the encoding process.

Sinogram squeezing can improve computational efficiency, but may cause pixel‐to‐pixel distortion. This distortion can occur because the values of neighboring pixels in the squeezed sinogram may be deformed differently from the values of the original filtered sinogram. In order to mitigate this issue, sinogram unfolding is conducted prior to sinogram squeezing. Sinogram unfolding concatenates the value of 3×3 neighboring pixels in the filtered sinogram, which is expressed as:

(6) $${\hat{z}}_{m,n}=\mathrm{Concat}\left({\left\{z_{m+i,n+j}\right\}}_{i,j\in -1,0,1}\right)$$

where Concat denotes concatenation, and the output unfolded sinogram $\hat{z}$ replaces the filtered sinogram and is used for sinogram squeezing. For simplicity, we will only represent the input of the encoder as z, regardless of whether unfolding is applied. Note that if unfolding is applied, since the number of channels in the squeezed sinogram is 9, the first 2D convolution layer in the encoder is set to a kernel of 1×1 size with nine input channels. Note that performing unfolding and using 1×1 convolution is equivalent to simply performing 3×3 convolution in an unsqueezed sinogram.

#### 3.2.2 Sinusoidal decoder

To ensure high image quality while increasing computational efficiency, we use a parameter‐free decoder instead of the MLP‐based decoder. It is inspired by OPE‐SR,^14^ which performs the ArbSR task for 2D photographic images using a set of sinusoidal bases that satisfies orthogonality. This decoder not only reduces inference and training time by not requiring decoder training but also has the advantage of being interpretable and thus more robust than conventional MLP‐based decoders. In this study, a set of sinusoidal bases is utilized for the 1D interpolation during back‐projection, and conversion into sinusoidal bases is performed using positional encoding as follows:

(7) $$\begin{array}{ccc} X & = & PE\left(x\right)=\sqrt{2}\cdot \left[\frac{1}{\sqrt{2}},\cos\left(\pi x\right),\sin\left(\pi x\right),\cos\left(2\pi x\right),\right.\\ & & \left.\sin(2\pi x),\cdots ,\cos(n\pi x),\sin(n\pi x)\right] \end{array}$$

Let $e_{i}$ be the ith element of a row vector X, and $e_{i}$ satisfies the orthogonality property as follows:

(8) $$\frac{1}{2}\int_{-1}^{1}e_{i_{1}}e_{i_{2}}dx=\left\{\begin{array}{cc} 1 & \text{if}\;i_{1}=i_{2}\\ 0 & \text{otherwise.} \end{array}\right.$$

Based on this orthogonality, back‐projection can also be expressed as a linear combination of a set of sinusoidal bases. In detail, the decoding function $f(z_{i}^{*},x_{iq}-x_{i})$ can be expressed as the inner product of the encoder feature and a set of sinusoidal bases as follows:

(9) $$\begin{array}{ccc} & & f\left(z_{i}^{*},x_{iq}-x_{i}\right)=z_{i}^{*}\cdot PE\left(x_{iq}-x_{i}\right),\\ & & \;\text{where}\;z_{i}^{*},PE\left(x_{iq}-x_{i}\right)\in R^{1\times n} \end{array}$$

Note that $z^{*}$ is the result of an additional convolution operation performed after encoding, as shown in the Figure 4, to ensure that the number of feature channels matches the number of sinusoidal bases. Additionally, the higher n values of the basis components are responsible for reconstructing the high‐frequency components on the image. However, due to the Nyquist–Shannon sampling theorem, a higher number of decoder bases does not always guarantee high‐quality image reconstruction.

### 3.3 Step‐2: Restoration module

Although the reconstruction module proposed above is expected to yield superior image quality compared to the FBP algorithm, residual artifacts and blurring remain, which degrade the diagnostic performance. Both remaining limitations are particularly pronounced at higher detector binning scales, where high‐frequency information is severely degraded. The poor visual quality due to the blurring and residual artifacts can be improved through additional post‐processing. As the reconstruction module exclusively utilizes sinogram domain data, the reconstructed image can be used for further image domain‐based post‐processing.

Therefore, we added the restoration module to perform additional post‐processing after the reconstruction module, as shown in the green shaded part of Figure 4. The restorator model removes blurring and residual artifacts that may remain in the Step‐1 result image, which can be applied optionally as needed. This enables a clearer representation of anatomical structures that may be difficult to visualize only using Step‐1. To train this restorator, we use a pretrained reconstruction module to perform transfer learning. This two‐step training strategy not only improves image quality but also provides more robustness than single‐step approaches that only use image domain data for restoration. Thanks to this robustness, our two‐step structure outperforms single‐step approaches especially when the binning scale is out of the range of the training dataset.

## 4 EXPERIMENT

### 4.1 Datasets

We used 1mm slice thickness normal‐dose CT image data from the “2016 NIH‐AAPM‐Mayo Clinic Low‐Dose CT Grand Challenge” dataset supported and approved by American Association of Physicists in Medicine (AAPM) and Mayo Clinic (AAPM‐Mayo).^38^ Sinograms are acquired using forward projection with additional Poisson noise by setting the number of incident photons in the projection to $1\;\times \;10^{6}$. For the 10 patients that comprise this dataset, 4, 482, 318, and 1136 image slices from 7, 1, and 2 patients are utilized as the train, validation, and test datasets, respectively. In addition, we utilize additional 1593 image slices from three patients from the clinical proteomic tumor analysis consortium sarcomas (CPTAC‐SAR) dataset^39^ as a test dataset. Detailed information about the FBCT geometry used for dataset preparation can be found in the Supplementary Material.

### 4.2 Training details

For training, the detector binning scale s is randomly selected from refs. [1, 2, 4], and for sinogram squeezing, the zero padding size and the expanding value d are set to 256/s and 64/s, respectively. This sinogram squeezing condition ensures that all the information is included in the squeezed sinogram to reconstruct a 128×128 size patch region in the GT image. For the Step‐1 encoder, we use the enhanced deep super‐resolution network (EDSR)‐baseline^40^ and residual dense network (RDN)^41^ without the upsampling module as in previous works. The encoders are trained for 300 epochs with a batch size of 4, and the initial learning rate is set to $1\times 10^{-4}$ and decays by 0.5 at [60, 120, 180, 240]. Next, for the Step‐2 restorator, SwinIR^42^ is trained for 300 epochs with a batch size of 4. The initial learning rate is set to $2\;\times \;10^{-4}$ and is decayed by 0.5 at [120, 180, 240, 270]. Both reconstruction and restoration modules employed in this study are optimized using supervised training, wherein the objective is to minimize the discrepancy between the output and the GT image, as illustrated in Figure 4. In detail, both modules are trained using Adam optimizer, and the L1 loss function is employed as the loss function. Moreover, since sinogram domain data requires a different approach for data augmentation, thus we optimized the data augmentation procedures. This data augmentation procedure and other details are in the Supplementary Material.

### 4.3 Details for image quality evaluation

We conduct a qualitative image evaluation of the resulting images from conventional techniques and the proposed technique. We focus our qualitative evaluation on mainly two cases: when the reconstruction resolution is fixed the same as the GT image at high binning scales such as ×4 and ×8, and when the reconstruction resolution varies without detector binning. This allows us to evaluate the superiority of the sinusoidal decoder structure of CRET.

For quantitative evaluation, peak signal‐to‐noise ratio (PSNR) and structural similarity index measure (SSIM) are used for quantitative evaluation metrics. Both metrics compare the error between the output image and the GT image and give a numerical indication of how close it is to the GT image.

**FIGURE 6:**
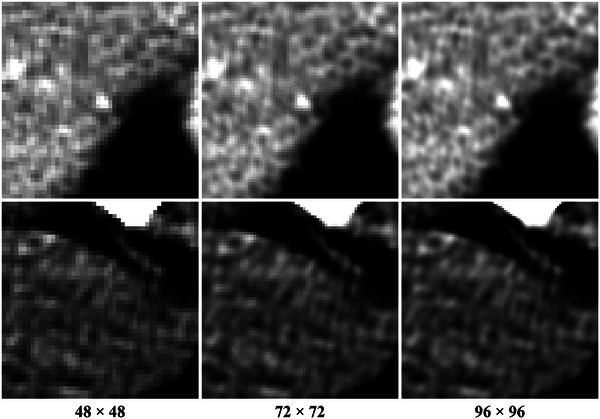
Example images of datasets configured for training and evaluating the observer model. Top row: Gaussian lesion present cases at the center. Bottom row: examples for lesion absent cases.

In addition, we use numerical observer performance for the lesion detection task. This compensates for the limitation that the calculation of quantitative metrics is intractable when the reconstruction resolution is higher than GT images. Furthermore, it enables evaluations correlated with diagnostic ability. In this study, we evaluated the performance of a lesion detection task under a signal‐known‐exactly (SKE) condition. This detection task classifies whether a lesion is present or absent in the center of the patch image. Example images are shown in Figure 6, and datasets are generated for each different reconstruction resolution.

Image slices from three patients in the CPTAC‐SAR dataset are utilized, and random region patches of size 48×48 are selected from the GT image for the background, excluding the air‐filled regions. For the lesion present cases, sinograms are generated with a projection of a Gaussian lesion with a standard deviation value of 0.75 and a peak value of 100 HU located in the center of the random region. We generated 75 000 data pairs in total: 50 000 lesion present and absent pairs and 25 000 pairs are used for training and test datasets, respectively.

For the observer model, we use difference‐of‐Gaussian channelized Hotelling observer (DOGCHO)^43^ to perform the detection task‐based quality evaluation. The DOGCHO is an anthropomorphic observer model that utilizes a channel matrix to limit detection performance to the level of the human visual system. This enables an investigation of the impact on lesion detection performance as reconstruction resolution changes from the perspective of the human visual system. In detail, the parameters of dense‐of‐Gaussian (DOG) channels are set to be identical to the settings of Abbey and Barrett^43^ For evaluation metrics, we compute the percent correct ($P_{c}$) value for evaluation. We estimated the $P_{c}$ value using bootstrapping output statistics 10 000 times.

## 5 RESULTS

### 5.1 Qualitative evaluation

**FIGURE 7:**
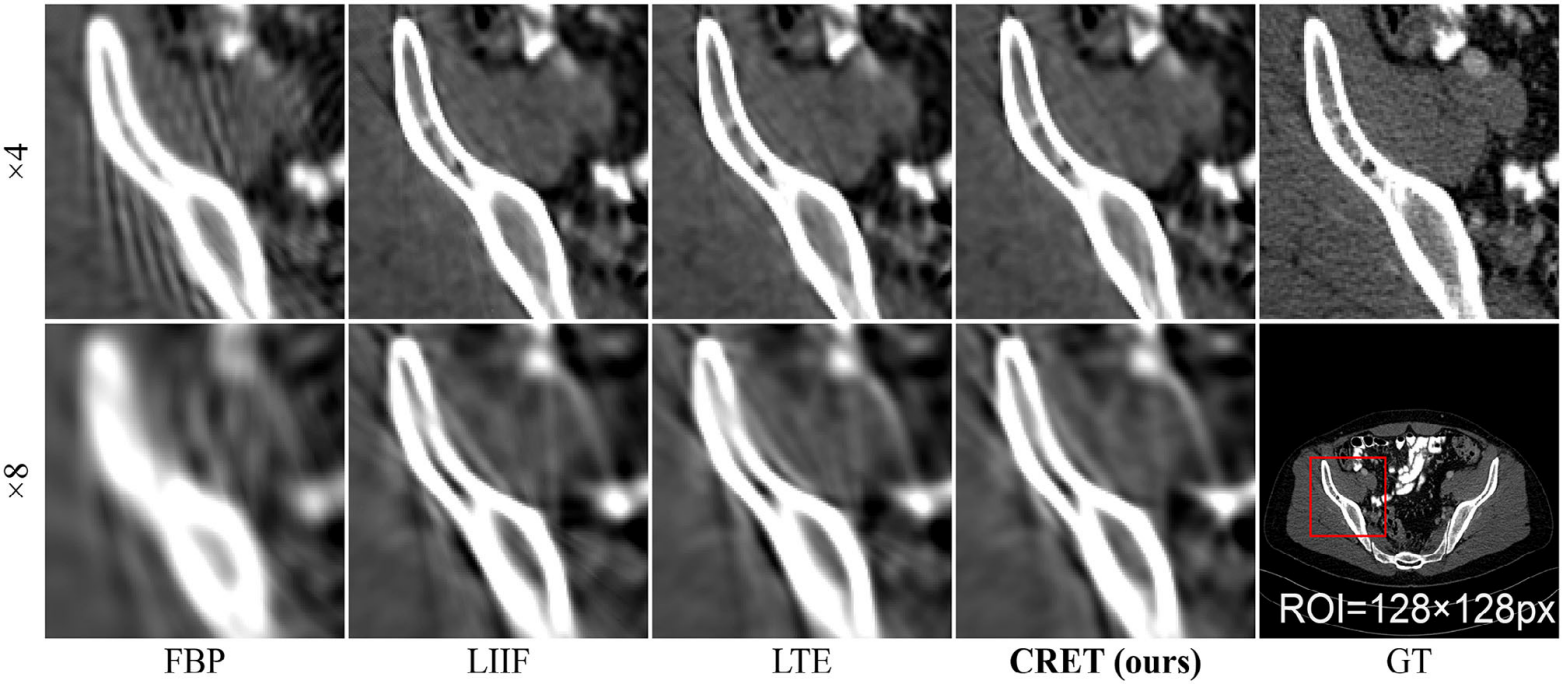
Qualitative evaluation of conventional methods using RDN^41^ as an encoder for high binning scales. The display window is [–120, 480] HU. HU, Hounsfield unit.

In the case of binning scales set to ×4 and ×8 as shown in Figure 7, the FBP algorithm generates severe artifacts throughout the reconstructed image. Although introducing LIIF into FBP can improve image quality, some artifacts and distortion still remain, particularly around structures with high attenuation coefficients such as bones. This tendency becomes more pronounced as the binning scale increases, especially with the ×8 binning scale, which is out of the range of scales used in the training dataset. While local texture estimator (LTE) can effectively reduce artifact and distortion issues compared to LIIF, some artifacts still remain. This limitation frequently arises due to the MLP‐based decoding mechanisms within local implicit representation‐based techniques, which remain challenging to explain due to the lack of interpretability of MLP‐based decoders. In contrast, our proposed CRET shows superior performance in terms of artifact and distortion mitigation compared to techniques using the MLP decoder.

**FIGURE 8:**
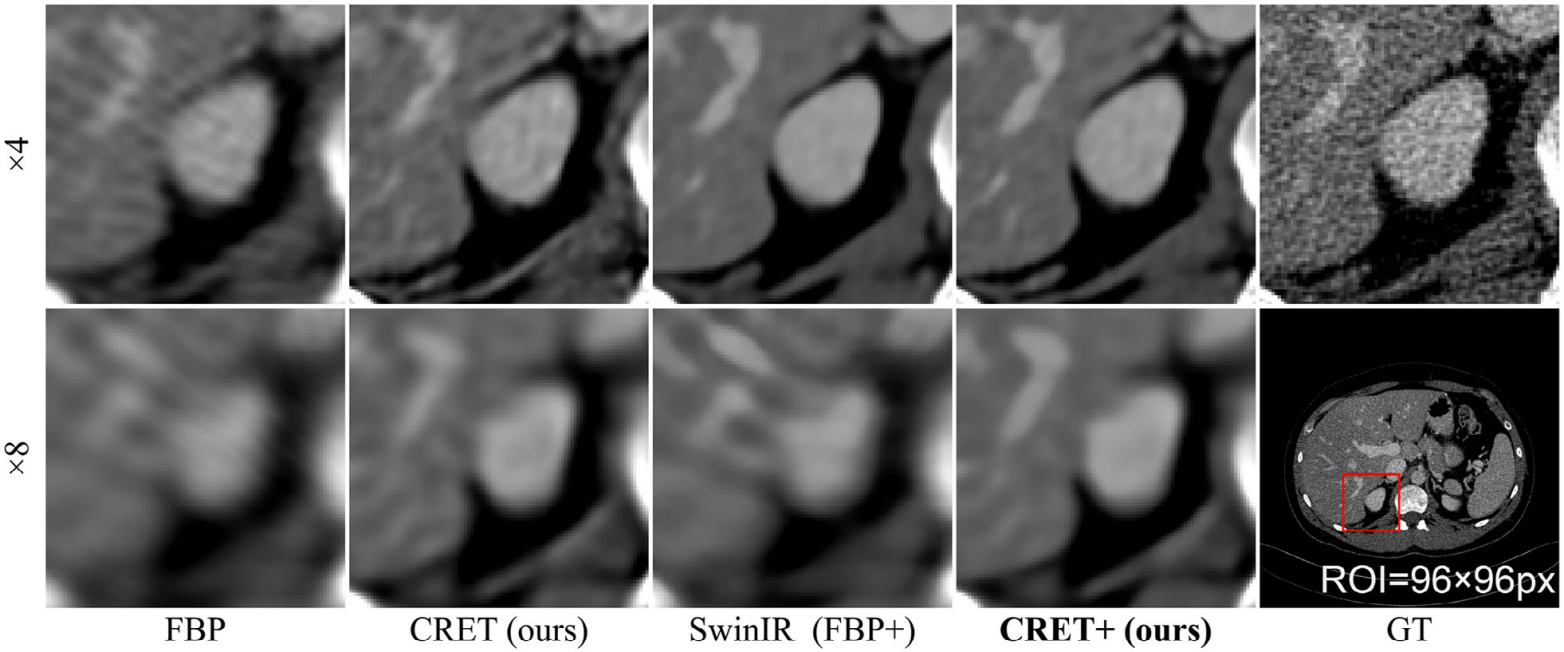
Qualitative assessment of applying restoration module for high binning scales. RDN^41^ is used for an encoder. The display window is [–80, 420] HU. HU, Hounsfield unit.

Next, Figure 8 compares the results for using the restoration module. Although CRET outperforms conventional local implicit representation methods in both quantitative and qualitative evaluations, residual artifacts, and blurring are still present in the reconstructed image. Both limitations can be addressed by CRET+, which further improves image quality through the restoration module. In particular, at the ×8 binning scale, CRET+ reduces artifacts and reconstructs the overall structure better than SwinIR, which uses the FBP image as an input. (as the simple FBP algorithm serves as the reconstruction module, this approach can be considered equivalent to FBP+).

**FIGURE 9:**
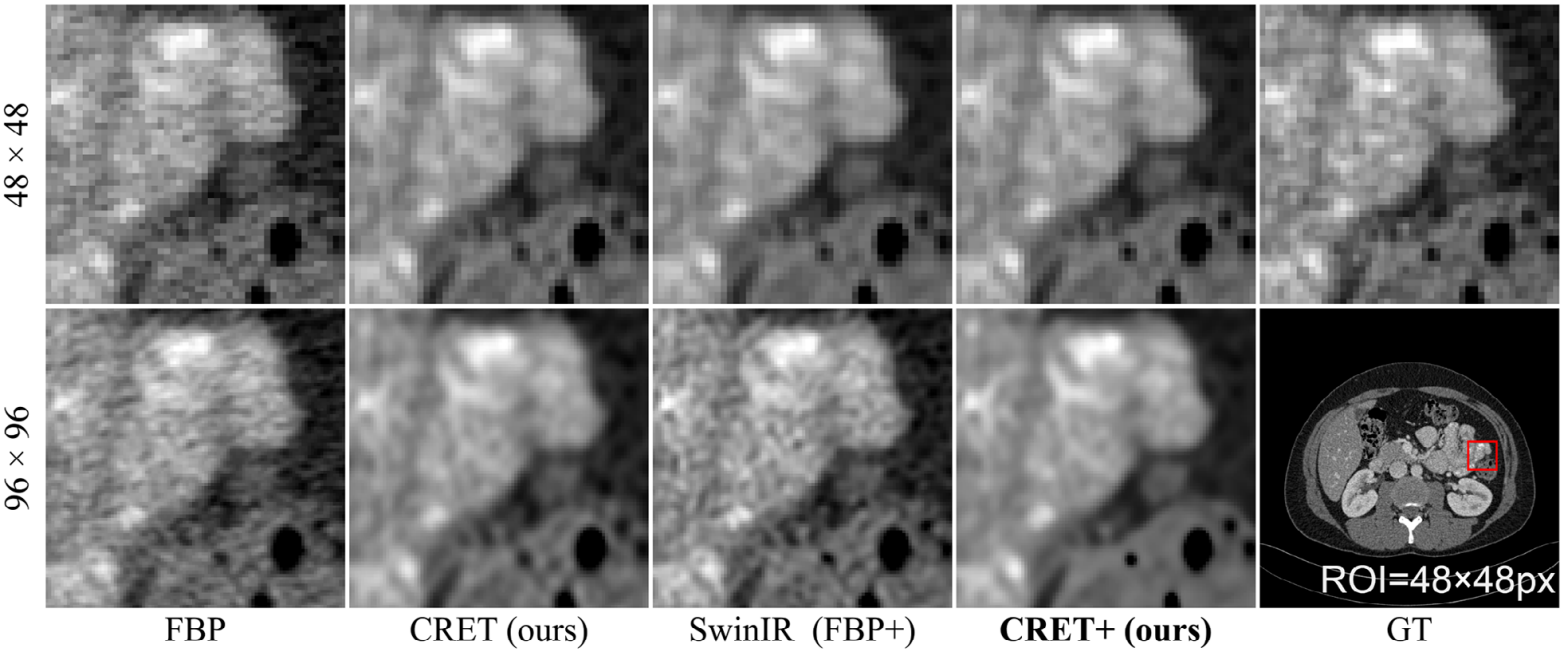
Qualitative assessment of different reconstruction resolutions when detector binning scale is ×1. RDN^41^ is used for the encoder, and the display window is [–180, 400] HU. HU, Hounsfield unit.

Furthermore, our proposed CRET also demonstrates outperforming quality when the reconstruction resolution exceeds that of the GT image as shown in Figure 9. When a radiologist uses a zoomed ROI image for the diagnosis of detailed anatomical structures, the limited array size of the ROI image hinders the diagnosis of the details. To address this problem, reconstruction resolution can be increased, which is preferred by radiologists.^44^ However, in the case of FBP, unresolved noise issue hinders accurate diagnosis. This issue cannot be mitigated by simply utilizing SwinIR in the image domain data because noise distribution is different from the training dataset. On the contrary, CRET demonstrates superior image quality regardless of the reconstruction resolution by reducing noise in the sinogram domain before reconstruction. Moreover, CRET+ further sharpens and corrects the image to improve its quality regardless of the image resolution. In addition, we also provide result images for more cases in the Supplementary Material.

### 5.2 Quantitative evaluation

Tables 1 and 2 shows a quantitative evaluation of PSNR and SSIM for different detector binning scales on the AAPM‐Mayo and CPTAC‐SAR test datasets, respectively. Note that Meta‐SR, LIIF, LTE, and CRET utilize sinogram data as input, SwinIR utilizes FBP images as input, and CRET+ utilizes both sinogram data and image data as input for the reconstruction and restoration module, respectively. First, utilizing sinogram domain data yields better quantitative results than FBP. However, structures that integrate representation techniques by incorporating an MLP‐based decoder are inferior to the decoder‐less methods that solely utilize an encoder. This is because decoder‐less methods are trained separately for each binning scale due to the lack of its generalizability, making them advantageous for quantitative evaluation. Furthermore, the lack of robustness of MLP‐based decoders causes performance degradation, which is another reason that decoder‐less methods are superior. In contrast, our proposed CRET achieves comparable or superior performance to decoder‐less methods overall. This is in contrast to the case of photographic image upsampling, where the sinusoidal decoder cannot outperform LIIF and LTE in terms of PSNR.^14^ This demonstrates that the interpretability and robustness of sinusoidal decoders have a positive impact on the image quality metric over MLP decoders for CT image reconstruction.

**TABLE 1:** Quantitative evaluation for different detector binning scales using the AAPM‐Mayo dataset with PSNR (dB) and SSIM.

	In‐scale			Out‐scale
Method	×1	×2	×4	×8
FBP	41.58 / 0.9738	37.22 / 0.9705	30.72 / 0.9156	26.81 / 0.8516
EDSR	46.25 / 0.9881	**44.44** / **0.9834**	**39.77** / **0.9671**	—
EDSR‐Meta‐SR	46.21 / 0.9881	44.22 / 0.9830	39.34 / 0.9654	31.08 / 0.9109
EDSR‐LIIF	**46.30** / 0.9881	44.19 / 0.9830	39.32 / 0.9653	31.20 / 0.9130
EDSR‐LTE	46.28 / 0.9882	44.29 / 0.9832	39.57 / 0.9664	31.34 / 0.9162
EDSR‐CRET (ours)	46.28 / **0.9883**	44.32 / 0.9833	39.72 / 0.9668	**31.52** / **0.9162**
RDN	46.38 / 0.9883	**44.65** / 0.9837	**40.31** / **0.9691**	—
RDN‐Meta‐SR	46.38 / 0.9883	44.40 / 0.9834	39.92 / 0.9676	31.40 / 0.9152
RDN‐LIIF	46.42 / 0.9883	44.39 / 0.9834	39.86 / 0.9674	31.53 / 0.9169
RDN‐LTE	46.41 / 0.9883	44.50 / 0.9836	40.18 / 0.9685	31.66 / 0.9196
RDN‐CRET (ours)	**46.45** / **0.9884**	44.55 / **0.9837**	40.28 / 0.9689	**31.83** / **0.9197**
SwinIR	46.57 / 0.9883	44.74 / 0.9835	40.15 / 0.9686	27.10 / 0.8516
EDSR-LIIF+	46.56 / 0.9883	44.79 / 0.9838	40.68 / 0.9704	31.36 / 0.9159
EDSR-LTE+	46.58 / 0.9884	44.86 / 0.9839	40.79 / 0.9709	31.42 / 0.9182
EDSR‐CRET+ (ours)	**46.64** / **0.9885**	**44.92** / **0.9840**	**40.98** / **0.9715**	**31.75** / **0.9200**
RDN-LIIF+	46.58 / 0.9885	44.84 / 0.9840	40.89 / 0.9712	31.74 / 0.9200
RDN-LTE+	46.65 / 0.9885	44.93 / 0.9841	41.09 / 0.9719	31.81 / **0.9222**
RDN‐CRET+ (ours)	**46.70** / **0.9886**	**44.99** / **0.9842**	**41.19** / **0.9722**	**31.96** / 0.9220

*Note*: The best results are highlighted as **bold**, and the second‐best results are highlighted as underline.

Abbreviations: LIIF, local implicit image function; PSNR, peak signal‐to‐noise ratio; SSIM, structural similarity index measure.

**TABLE 2:** Quantitative evaluation for different detector binning scales using the CPTAC‐SAR dataset with PSNR (dB) and SSIM.

	In‐scale			Out‐scale
Method	×1	×2	×4	×8
FBP	40.95 / 0.9694	36.67 / 0.9652	31.43 / 0.9232	27.59 / 0.8754
EDSR	44.70 / 0.9841	**42.96** / **0.9799**	38.84 / 0.9664	—
EDSR‐Meta‐SR	44.68 / 0.9840	42.62 / 0.9793	38.38 / 0.9645	31.32 / 0.9162
EDSR‐LIIF	45.06 / 0.9843	42.72 / 0.9795	38.35 / 0.9646	31.49 / 0.9185
EDSR‐LTE	44.91 / 0.9842	42.83 / 0.9796	38.70 / 0.9659	31.50 / 0.9202
EDSR‐CRET (ours)	**45.12** / **0.9844**	42.94 / 0.9798	**38.95** / **0.9665**	**31.67** / **0.9205**
RDN	44.83 / 0.9843	43.14 / **0.9804**	39.53 / 0.9686	—
RDN‐Meta‐SR	45.04 / 0.9844	43.04 / 0.9800	39.08 / 0.9671	31.60 / 0.9200
RDN‐LIIF	45.24 / 0.9845	42.99 / 0.9800	39.03 / 0.9670	31.71 / 0.9216
RDN‐LTE	45.06 / 0.9844	43.05 / 0.9800	39.35 / 0.9680	31.87 / 0.9240
RDN‐CRET (ours)	**45.42** / **0.9847**	**43.24** / 0.9802	**39.65** / **0.9688**	**31.94** / **0.9241**
SwinIR (FBP+)	43.44 / 0.9837	42.60 / 0.9797	38.26 / 0.9656	27.82 / 0.8744
EDSR-LIIF+	45.12 / 0.9844	43.17 / 0.9803	39.27 / 0.9688	31.60 / 0.9205
EDSR-LTE+	45.23 / 0.9845	43.34 / 0.9806	39.53 / 0.9697	31.60 / 0.9218
EDSR‐CRET+ (ours)	**45.30** / **0.9847**	**43.44** / **0.9807**	**39.85** / **0.9705**	**31.88** / **0.9235**
RDN-LIIF+	45.37 / 0.9847	43.39 / 0.9808	39.69 / 0.9702	31.81 / 0.9235
RDN-LTE+	45.43 / 0.9847	43.50 / 0.9808	39.86 / 0.9708	32.02 / **0.9263**
RDN‐CRET+ (ours)	**45.54** / **0.9849**	**43.60** / **0.9809**	**40.16** / **0.9714**	**32.07** / 0.9257

*Note*: The best results are highlighted as **bold**, and the second‐best results are highlighted as underline.

Abbreviations: FBP, filtered back‐projection; LIIF, local implicit image function; PSNR, peak signal‐to‐noise ratio; SSIM, structural similarity index measure.

Next, we compare techniques that utilize image data as an input for the restoration module. As a result, using a Step‐1 result image as a restorator input achieves higher image quality than simply using an FBP image as an input for the restorator. In particular, in the case of detector binning scale is ×8 which is out of the range of scales used in training, SwinIR cannot improve quality much over FBP. On the other hand, our proposed CRET+ significantly improves the quality of FBP.

### 5.3 Observer evaluation

**FIGURE 10:**
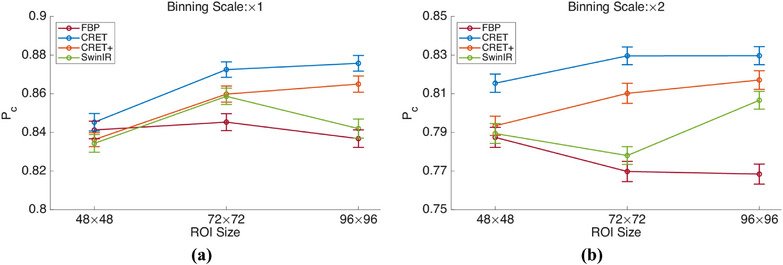
Plots of confidence interval of $P_{c}$ values for different reconstruction resolution of ROI patch. (a) and (b) show the case where the detector binning scale is set to ×1 and ×2, respectively. ROI, region of interest.

As shown in Figure 10, significant performance improvement is not observed by increasing the resolution in the case of FBP. This is because when the binning scale is ×1, even if the resolution is increased, the remaining noise still hinders lesion detection. Furthermore, the noise reduction resulting from detector binning cannot improve lesion detection performance, as the blurring effect may hinder the lesion detection. On the other hand, CRET mitigates the impact of noise and blurring, and significant improvements in detection performance are observed with increasing resolution for both the ×1 and ×2 cases.

Next, SwinIR, which utilizes only the image domain data, shows a limited performance improvement overall. On the contrary, CRET+, which utilizes the output image obtained from CRET as the SwinIR input, shows a significant performance improvement. However, although CRET+ outperforms CRET in terms of quantitative and qualitative evaluation, lower detection performances are observed. This tendency has also been observed in channelized hotelling observer (CHO)‐based observer evaluations in other studies,^45^, ^46^ suggesting that an additional image domain restoration step is unlikely to improve lesion detection performance under SKE conditions. Note that these tendencies can differ if evaluations are performed under different scenarios, such as a signal‐known‐statistically (SKS) condition. In addition, the restoration module is a plug‐and‐play design, empowering radiologists to decide on its application based on their needs. Thanks to this plug‐and‐play feature, radiologists can fine‐tune results for optimal visualization in diverse clinical scenarios.

### 5.4 Computing time and memory efficiency

We evaluate memory and computing efficiency with memory consumption, floating point operations (FLOPs), and running time. For evaluation, we used an NVIDIA RTX A6000 GPU for all experiments. In Table 3, LIIF and LTE require a large amount of time and memory due to the extensive size of the decoder input data. This tendency becomes more pronounced as the resolution increases, making it impractical to utilize LIIF and LTE for CT image reconstruction. In contrast, our proposed CRET uses only 8%−29% of memory compared to conventional methods and also requires only 16%−23% of runtime compared to other methods due to its high computational complexity efficiency. Even with the large reconstruction resolution condition, CRET is highly efficient, allowing radiologists to obtain result images almost immediately in practice. Note that this result is obtained without further reducing the decoder input data size or any optimizations for GPU hardware specifically applied to CRET. The superiority of our proposed CRET is achieved by simply reducing the decoding computation by using a parameter‐free sinusoidal decoder, as confirmed by the FLOPs results in column 4 of Table 3.

**TABLE 3:** Comparison for time complexity and memory consumption for the decoder of each method.

Recon size	Method	Mem	FLOPs	Runtime
64×64	LIIF	6.6 G	90.0 G	51.8 ms
	LTE	1.9 G	46.6 G	65.6 ms
	CRET	0.5 G	1.1 G	12.0 ms
128×128	LIIF	26.2 G	359.8 G	205.6 ms
	LTE	7.4 G	157.4 G	253.7 ms
	CRET	2.0 G	1.9 G	40.5 ms

*Note*: Recon size, resolution of the reconstructed ROI image; Mem, allocated GPU memory; FLOPs, floating point operations; Runtime, execution time for an ROI image reconstruction.

Abbreviations: FLOPs, floating point operations; LIIF, local implicit image function.

### 5.5 Ablation studies

#### 5.5.1 Analysis of the number of sinusoidal bases

To investigate the impact of the number of sinusoidal bases in the reconstruction module on performance, we train each reconstruction module with different numbers of sinusoidal bases n and evaluate the PSNR for each case. The evaluation results are shown in Table 4, which shows the PSNR and runtime as the n value varies. For low values of n, performance degrades at high binning scales due to the poor representation ability of high‐frequency components. However, using a high n value does not guarantee a positive impact on the PSNR value and is more time‐consuming as mentioned in the previous section. Based on this result, we set the value of n to five for all CRET and CRET+ structures used in this study. Moreover, further discussions and analysis are included in the Supplementary Material.

**TABLE 4:** Analysis as the number of decoding bases n varies.

	In‐scale			Out‐scale
n	×1	×2	×4	×8
1	46.26 / 36.4	44.28 / 29.4	39.66 / 25.2	31.42 / 22.7
3	46.27 / 45.7	44.32 / 38.5	39.72 / 34.5	31.45 / 32.0
5	46.28 / 55.9	44.32 / 49.1	39.72 / 44.7	31.52 / 42.2
7	46.27 / 68.7	44.32 / 61.6	39.71 / 57.5	31.50 / 55.2

*Note*: PSNR (dB)/runtime for reconstructing 128×128 size ROI image (ms). EDSR‐baseline is used for the encoder and evaluated using the AAPM‐Mayo dataset.

Abbreviations: PSNR, peak signal‐to‐noise ratio; ROI, region of interest; Runtime, execution time for an ROI image reconstruction.

#### 5.5.2 Impact of the sinogram squeezing and unfolding

Table 5 shows the results for whether sinogram squeezing and unfolding are applied. In the case where sinogram squeezing is not applied, even if reconstruction is performed for a specific ROI, the entire sinogram is required, which shows high computational complexity and runtime. On the other hand, applying sinogram squeezing reduces computational complexity and execution runtime. Also, it improves image quality significantly for low binning scale cases, but sacrificing quality for high binning scale cases. In summary, applying sinogram squeezing can result in a trade‐off between runtime and PSNR for high binning scales. To address this trade‐off dilemma, sinogram unfolding can be utilized. Simply performing the unfolding before the sinogram squeezing improves image quality in overall cases without increasing computational complexity.

**TABLE 5:** Impact of the sinogram squeezing (**S**) and unfolding (**U**) on the FLOPs, runtime, and PSNR (dB).

				In‐scale			Out‐scale
**S**	**U**	FLOPs	Runtime	×1	×2	×4	×8
✗	✗	462.0 G	86.0 ms	45.65 / 0.9873	44.17 / 0.9831	39.90 / 0.9676	31.49 / 0.9156
✓	✗	161.4 G	56.4 ms	46.36 / 0.9882	44.29 / 0.9832	39.58 / 0.9664	31.41 / 0.9153
✓	✓	161.4 G	56.7 ms	46.28 / 0.9883	44.32 / 0.9833	39.72 / 0.9668	31.52 / 0.9161

*Note*: FLOPs and runtime are measured for reconstructing a 128×128 size ROI image without detector binning, and the runtime includes the time spent on sinogram squeezing. EDSR‐baseline is used for the encoder and evaluated using the AAPM‐Mayo dataset.

Abbreviations: FLOPs, floating point operations; Runtime, execution time for an ROI image reconstruction; PSNR, peak signal‐to‐noise ratio.

## 6 DISCUSSION

This work proposes a structure that integrates a continuous image representation technique into FBP for CT reconstruction, aiming to improve the limited spatial resolution of CT images. Our proposed CRET improves image quality significantly compared to other conventional methods, as evidenced by multiple evaluation metrics. We confirm that sinogram squeezing and sinusoidal decoding resolve the inherent limitations that require excessive computing resources. Specifically, the proposed sinogram squeezing demonstrates simply performing pre‐processing on the input sinogram data can improve encoding efficiency and output image quality. The EDSR and RDN architectures utilized as encoders in this study were originally developed for the SR of photographic images. Except for removing the pixel shuffle upsampling layer in the final stage, no other modifications were made to these architectures. While these encoder architectures are effective at feature extraction for photographic images, they are not fully optimized for the sinogram domain data, which shows different data distribution from that of photographic images. Consequently, encoding for feature extraction can be less efficient than in photographic imaging, resulting in limited reconstruction image quality. Future work could further optimize the encoder architecture for sinogram data to improve encoding efficiency, resulting in reduced computational resource requirements or improved reconstructed image quality.

Beyond blurring and noise addressed in this work, our proposed framework has the potential to be extended to mitigate other degradation factors such as streak artifacts, suggesting a generalized model for overall degradation factors. As demonstrated by our experiments, CRET significantly enhances spatial resolution while effectively mitigating noise and blurring across different detector binning scales. This robustness highlights its potential for addressing diverse CT imaging scenarios. However, if acquisition settings beyond binning scales differ, other effects not considered in this study are produced. For instance, adjusting the number of projection views allows for lower radiation dose; but introduces streak artifacts, which are different from noise and blurring effects mainly addressed in this study. To achieve high performance reconstruction that can generalize across multiple degradation factors, a novel encoder structure should be designed. This structure offers the capability of adapting to different CT scan settings, thereby providing a more flexible and robust structure.

Next, our proposed CRET has the potential to be further extended to other types of tomographic imaging. One of the key contributions of this work, sinogram squeezing, is inspired by the property that only a specific region of sinogram can be utilized for an ROI reconstruction. In addition, sinusoidal basis decoding utilized in this study can be applied to any back‐projection‐based reconstruction method that performs interpolation. Other types of tomographic imaging modalities share these properties, indicating that the proposed sinogram squeezing is not limited to FBCT geometry and can be extended to other geometries. For instance, interpolation process employed in the Feldkamp–Davis–Kress (FDK) algorithm^47^ for cone‐beam CT (CBCT) reconstruction is similar to the FBCT back‐projection step. By leveraging this similarity, our proposed CRET method can be utilized for CBCT reconstruction, by stacking multiple input axial projection data in a batch and computing them in parallel. However, this approach is effective only when the CBCT cone angle is small, and it is difficult to improve the resolution along the *z*‐axis, which is perpendicular to the axial plane. To mitigate these limitations, the encoder structure needs to be modified to suit increased projection data dimension. However, as the dimensional complexity of the input data increases, computational demands and memory usage increase exponentially, rendering it impractical. Therefore, to extend the proposed CRET to other geometries beyond FBCT, it is necessary to optimize the encoder structure according to the characteristics of the projection data used as input for a practical design.

Future work can evaluate more aspects beyond the model observer used in this study. In terms of detection performance from DOGCHO, our proposed CRET outperforms other methods. However, unlike the results of PSNR evaluation, CRET+, which performs an additional restoration step in the image domain, demonstrates worse results than CRET. As mentioned in the previous section, this tendency is also common in other studies and may show different trends when evaluated through other task‐based image quality metrics. For instance, evaluating image quality via edge detection or image segmentation performance, image‐domain‐based methods can have a positive impact.^48^ Moreover, although observer‐based quality assessment is regarded as a robust indicator of diagnostic ability, additional assessments, including a reader study with radiologists are required. Therefore, a multifaceted evaluation should be conducted to assess the impact of each module of the proposed CRET to determine whether it is practical to use.

## 7 CONCLUSION

We propose CRET, a novel structure that enables visualizations that exceed the limited spatial resolution of conventional CT imaging, thereby helping the diagnosis of anatomical details. The proposed CRET utilizes sinogram squeezing and a sinusoidal decoder to reduce computational time and memory consumption for image reconstruction. This addresses a major constraint of local implicit representation‐based methods, which require excessive computing resources for improving spatial resolution. In addition, we demonstrated that our CRET provides superior image quality compared to conventional techniques as well.

## CONFLICT OF INTEREST STATEMENT

The authors declare no conflicts of interest.

## Supporting information

Supporting Information

Supporting Information

Supporting Information
