# Supplementary material for: Continuous representation‐based reconstruction for computed tomography
Source: Med Phys. 2025 May 2;52(7):e17849. doi: 10.1002/mp.17849 (PMC12257912; doi:10.1002/mp.17849)
Supplement: Supplementary file 1 — Supporting Information [file MP-52-0-s002.pdf]

I. Appendix A: Experiment Details

I.A. Parameters of FBCT geometry

In this work, forward-projection and back-projection are performed in an equiangular FBCT geometry as previously expressed. The forward-projection and back-projection are performed on the same FBCT geometry for all patients data. The parameter values for the FBCT geometry are listed in table A1. Note that some values such as the number of detectors and size of detector element can be changed based on variations in the detector binning scale $\mathbf{s}$. In addition, the same geometry values are also used for the test dataset, but if the reconstruction resolution differs, the pixel size can be adjusted.

I.B. Data Augmentation for Sinogram data

In the case of SR for photographic imaging, random 90° rotation, horizontal flip, and vertical flip are adapted to the LR and HR image pair for data augmentation in general. However, our proposed CRET method uses a sinogram as an input and produces a reconstructed image as an output. Therefore, since the data domains of input and output are different, a novel data augmentation technique is required that is different from conventional methods. In this study, we modified the data augmentation technique accordingly to take into account the relationship between the sinogram domain and the image domain data. This data augmentation technique between the input sinogram and the target image can be summarized as Algorithm 1. We applied this data augmentation technique to all experiments in this study.

Algorithm 1 Data Augmentation

Input: sinogram $\mathbf{z} \in \mathbb{R}^{N \times D}$, image $\mathbf{y} \in \mathbb{R}^{H \times W}$
Output: augmented sinogram $\mathbf{z}' \in \mathbb{R}^{N \times D}$, augmented image $\mathbf{y}' \in \mathbb{R}^{H \times W}$
switch Augmentation **do**
  case Rotation 90°
    $\mathbf{z}' = \textbf{Concatenate}(\mathbf{z}[N/4 ::, :], \mathbf{z}[: N/4, :])$
    $\mathbf{y}' = \textbf{Rotate}90^\circ(\mathbf{y})$
  case Horizontal Flip
    $\mathbf{z} = \textbf{HorizontalFlip}(\mathbf{z})$
    $\mathbf{z}' = \textbf{Concatenate}(\mathbf{z}[0, :], \mathbf{z}[: 0 : -1, :])$
    $\mathbf{y}' = \textbf{HorizontalFlip}(\mathbf{y})$
  case Vertical Flip
    $\mathbf{z} = \textbf{VerticalFlip}(\textbf{HorizontalFlip}(\mathbf{z}))$
    $\mathbf{z}' = \textbf{Concatenate}(\mathbf{z}[N/2 - 1 :, :], \mathbf{z}[: N/2 - 1, :])$
    $\mathbf{y}' = \textbf{VerticalFlip}(\mathbf{y})$

I.C. Implementation Details

In this study, Meta-SR[1], LIIF[2], LTE[3], and our proposed CRET are used for comparison among continuous representation-based techniques. Note that sinogram squeezing is also applied to CRET as well as all other techniques to reduce training time and memory consumption. Unlike photographic images where the corresponding LR patch image can be used when the target is a specific ROI patch, CT imaging requires the entire sinogram for filtering purposes. Accordingly, we apply the same detector binning scale to the entire sinogram batch and perform training using the filtered sinogram batch.

In particular, LIIF-based local implicit representation methods require excessive decoding time and memory consumption, making training and inference difficult in practice. To address this issue, we sample the input patch for training local implicit representation methods at a size of 48×48, similar to the training photographic image ArbSR task. For decoding layer, we employ a 3-hidden-layer MLP with 128 hidden dimensions for LIIF and a 2-hidden-layer MLP with with 128 hidden dimensions for LTE.

Next, we conduct a comparative study by adding a restoration module to each of the following methods when utilized as Step-1: LIIF, LTE, and our proposed CRET. Note that applying additional restoration module after LIIF and LTE is not practical due to the excessive time and memory consumption. Consequently, these approaches lead to inefficiency in both the training and inference processes, making implementation challenging in our GPU environment. As a result, training conditions such as batch size are not identical for CRET+.

In addition, we perform comparisons with simple decoder-less methods as a non-representational approach. These decoder-less methods perform image reconstruction using simple back-projection instead of a decoder, similar to the structure proposed by Wrfl et al.[4] for limited-angle reconstruction. However, a key difference is that this study addresses the case where detector binning is applied; therefore, upsampling should be performed prior to the back-projection to achieve higher image quality. Consequently, a sub-pixel upsampling layer was utilized in the end of the encoder, distinguishing it from the technique proposed by Wrfl et al.[4].

II. More Visual Comparisons

Figure A1 compares the results of the methods for cases of high detector binning scale and shows that the proposed CRET outperforms other representation-based methods. In addition, in the case of utilizing image data, our proposed CRET+ is better for restoring overall structures and removing artifacts than simply using SwinIR[5]. Next, figure A2 shows the results of varying the reconstruction resolution. Note that in all the resulting figures, RDN[6] is used as an encoder for representation-based methods.

References

[1] X. Hu, H. Mu, X. Zhang, Z. Wang, T. Tan, and J. Sun, Meta-SR: A magnification-arbitrary network for super-resolution, in *Proceedings of the IEEE/CVF conference on computer vision and pattern recognition*, pages 1575–1584, 2019.

[2] Y. Chen, S. Liu, and X. Wang, Learning continuous image representation with local implicit image function, in *Proceedings of the IEEE/CVF conference on computer vision and pattern recognition*, pages 8628–8638, 2021.

[3] J. Lee and K. H. Jin, Local texture estimator for implicit representation function, in *Proceedings of the IEEE/CVF conference on computer vision and pattern recognition*, pages 1929–1938, 2022.

[4] T. Würfl, M. Hoffmann, V. Christlein, K. Breininger, Y. Huang, M. Unberath, and A. K. Maier, Deep learning computed tomography: Learning projection-domain weights from image domain in limited angle problems, IEEE transactions on medical imaging **37**, 1454–1463 (2018).

[5] J. Liang, J. Cao, G. Sun, K. Zhang, L. Van Gool, and R. Timofte, Swinir: Image restoration using swin transformer, in *Proceedings of the IEEE/CVF international conference on computer vision*, pages 1833–1844, 2021.

[6] Y. Zhang, Y. Tian, Y. Kong, B. Zhong, and Y. Fu, Residual dense network for image super-resolution, in *Proceedings of the IEEE conference on computer vision and pattern recognition*, pages 2472–2481, 2018.

Table A1: Geometry parameters of equiangular FBCT for forward-projection and back-projection.

Parameter Name	Value
Distance between the source and detector center (mm)	1085.6
Distance between the source and isocenter (mm)	595
Number of view (number)	512
Number of detector elements (number)	$736/\mathbf{s}$
Size of each detector element (mm)	$1.2858 \times \mathbf{s}$
Pixel size of the reconstructed image (mm)	0.6641

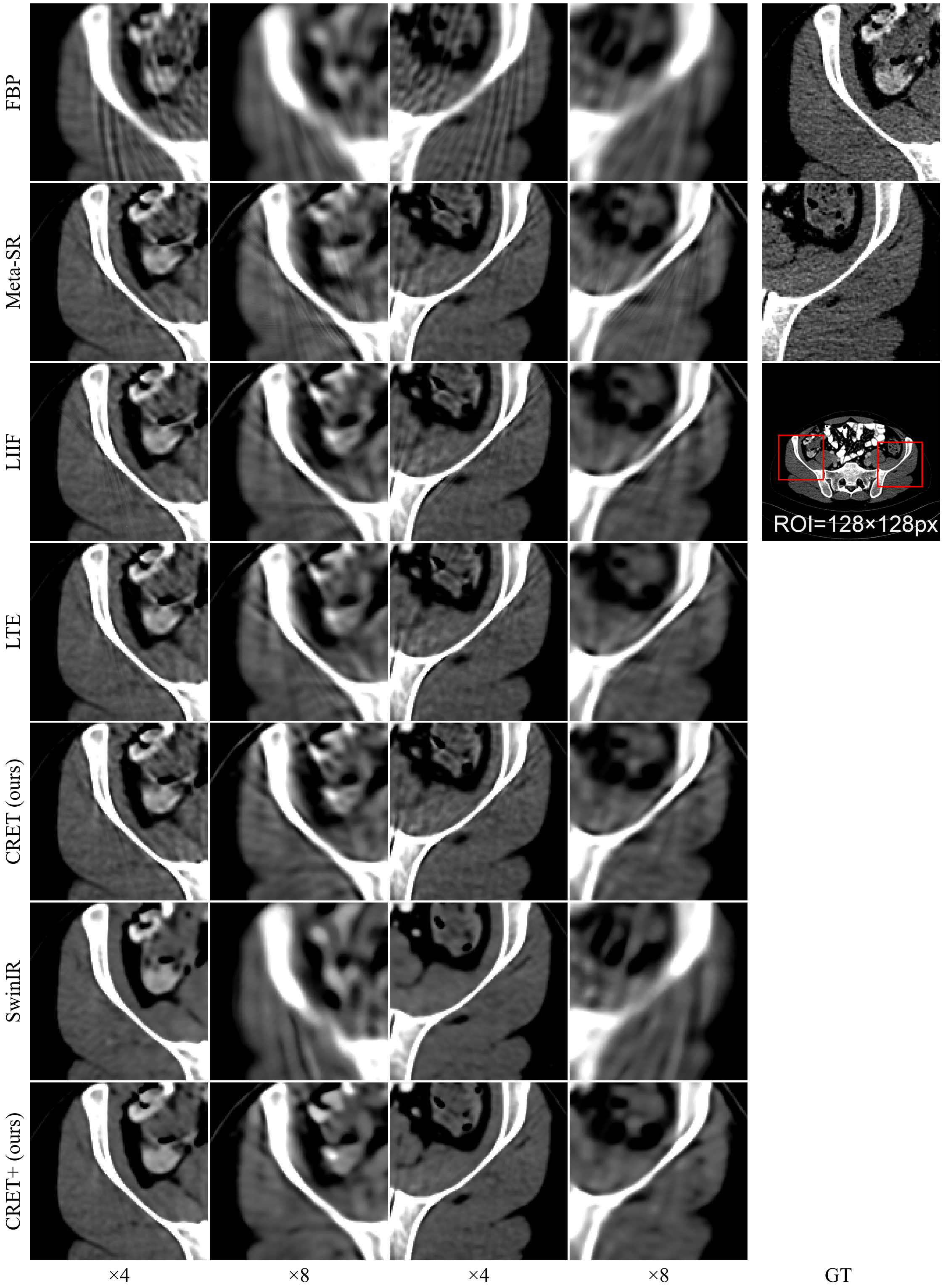

Figure A1: Comparison for high binning scale cases. The display windows are [-60, 320] HU.

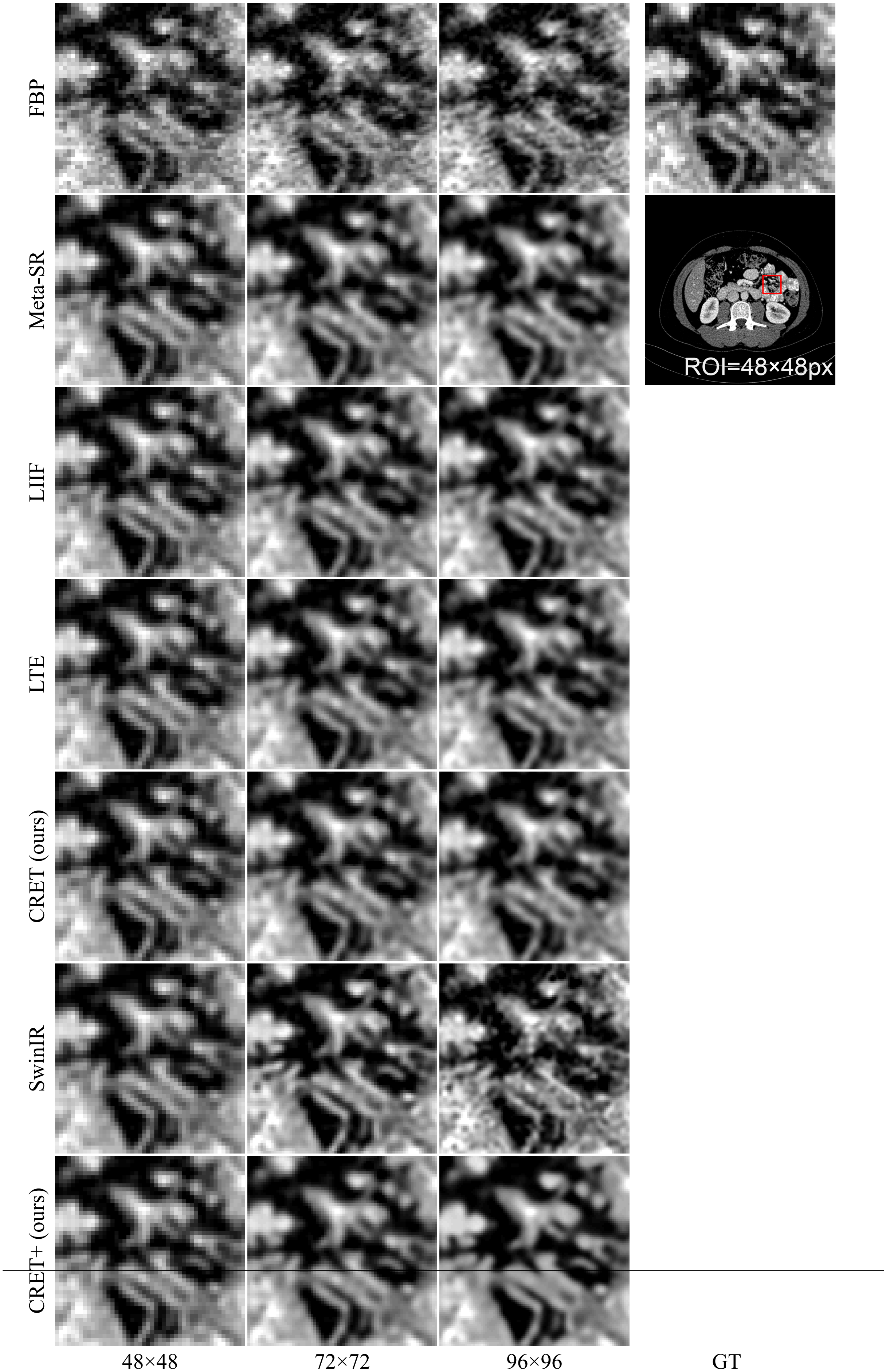
FBP
Meta-SR
LIIF
LTE
CRET (ours)
SwinIR
CRET+ (ours)
ROI=48×48px
48×48
72×72
96×96
GT
